# MicroRNA-106b-5p inhibits growth and progression of lung adenocarcinoma cells by downregulating IGSF10

**DOI:** 10.18632/aging.203318

**Published:** 2021-07-29

**Authors:** Bo Ling, Xianjiu Liao, Qiang Tang, Guangbin Ye, Xiaoyun Bin, Jianchu Wang, Yaqin Pang, Guangzi Qi

**Affiliations:** 1 College of Pharmacy, Youjiang Medical University for Nationalities, Baise 533000, Guangxi, China; 2 Department of Burn and Plastic Surgery and Wound Repair, Affiliated Hospital of Youjiang Medical University for Nationalities, Baise 533000, Guangxi, China; 3 College of Basic Medical Sciences, Youjiang Medical University for Nationalities, Baise 533000, Guangxi, China; 4 Medical College of Guangxi University, Nanning 530004, Guangxi, China; 5 Department of Hepatobiliary Surgery, Affiliated Hospital of Youjiang Medical University for Nationalities, Baise 533000, Guangxi, China; 6 College of Medical Laboratory, Youjiang Medical University for Nationalities, Baise 533000, Guangxi, China; 7 College of Public Health and Management, Youjiang Medical University for Nationalities, Baise 533000, Guangxi, China

**Keywords:** lung adenocarcinoma, IGSF10, prognosis, biomarker, miR-106b-5p

## Abstract

In this study, we investigated the mechanistic role and prognostic significance of IGSF10 in lung adenocarcinoma. Oncomine database analysis showed that IGSF10 expression was significantly reduced in most cancer types, including lung adenocarcinoma (LUAD). In the TCGA-LUAD dataset, IGSF10 expression correlated positively with proportions of tumor-infiltrated B cells, CD4^+^ T cells, CD8^+^ T cells, neutrophils, macrophages, and dendritic cells. Kaplan-Meier survival analysis showed that overall survival of patients with low IGSF10 expression was significantly shorter than those with high IGSF10 expression. MiRWalk2.0 database analysis and dual luciferase reporter assays confirmed that miR-106b-5p suppressed IGSF10 expression by binding to its 3’UTR. MiR-106b-5p levels inversely correlated with IGSF10 expression in the TCGA-LUAD dataset. Moreover, inhibition of miR-106b-5p significantly decreased *in vitro* proliferation, migration, and invasion by LUAD cells, whereas miR-106b-5p overexpression reversed those effects. These results demonstrate that IGSF10 is an independent prognostic factor for LUAD. Furthermore, miR-106b-5p suppressed IGSF10 expression in LUAD tissues by binding to its 3’UTR, which makes IGSF10 and miR-106b-5p potential prognostic biomarkers and therapeutic targets in LUAD patients.

## INTRODUCTION

Lung cancer is the leading cause of tumor-related deaths worldwide [1]. The two main types of lung cancer are non-small cell lung cancer (NSCLC) and small cell lung cancer (SCLC), with NSCLC accounting for 85% of all lung cancer cases worldwide [2]. Despite advances in treatment strategies in the recent years, the prognosis of NSCLC patients is poor because most lung cancer patients are diagnosed in the advanced stages and are associated with high rates of regional and distant metastases [3, 4]. Therefore, new and more effective treatment strategies are urgently required to improve the prognosis of NSCLC patients.

Molecular mechanisms that regulate embryonic development also play a key role in tumorigenesis [5, 6]. Furthermore, advanced and aggressive cancers demonstrate abnormal expression of developmental genes [7, 8]. In our previous study, we demonstrated that IGSF10 played a tumor suppressor role in lung cancer and was a potential prognostic factor for lung cancer patients [9]. IGSF10 is a member of the immunoglobulin super-family that regulates early migration of GnRH (gonadotropin-releasing hormone) neurons and their differentiation. Abnormal expression of IGSF10 also contributes to developmental delays and delayed puberty [10, 11]. However, the regulatory role of IGSF10 in lung cancer remains unclear.

MicroRNAs (miRNAs) are single-stranded non-coding RNAs of about 20-24 nucleotides in length that bind to the 3’-UTR of their target mRNAs and suppress translation [12]. They regulate expression levels of their target oncogenes or tumor suppressor genes, and are promising targets for cancer diagnosis and treatment [13–15]. MiRNA-106b-5p is located on chromosome 7q21 and plays either oncogenic or tumor suppressor functions in various cancers [16–18]. MiR-106b-5p promotes proliferation of NSCLC cells by targeting BTG3 [19]. Moreover, miR-106b-5p regulates cisplatin chemosensitivity in NSCLC via PKD2 [20]. The relationship between miR-106b-5p and IGSF10 in NSCLC is not reported. Therefore, in this study, we performed bioinformatics analysis and *in vitro* experiments to determine the prognostic significance and mechanistic role of IGSF10 in lung adenocarcinoma (LUAD). We also investigated the relationship between IGSF10 and miR-106b-5p in lung adenocarcinoma (LUAD).

## RESULTS

### IGSF10 expression is significantly reduced in lung cancer tissues

**Figure 1:**
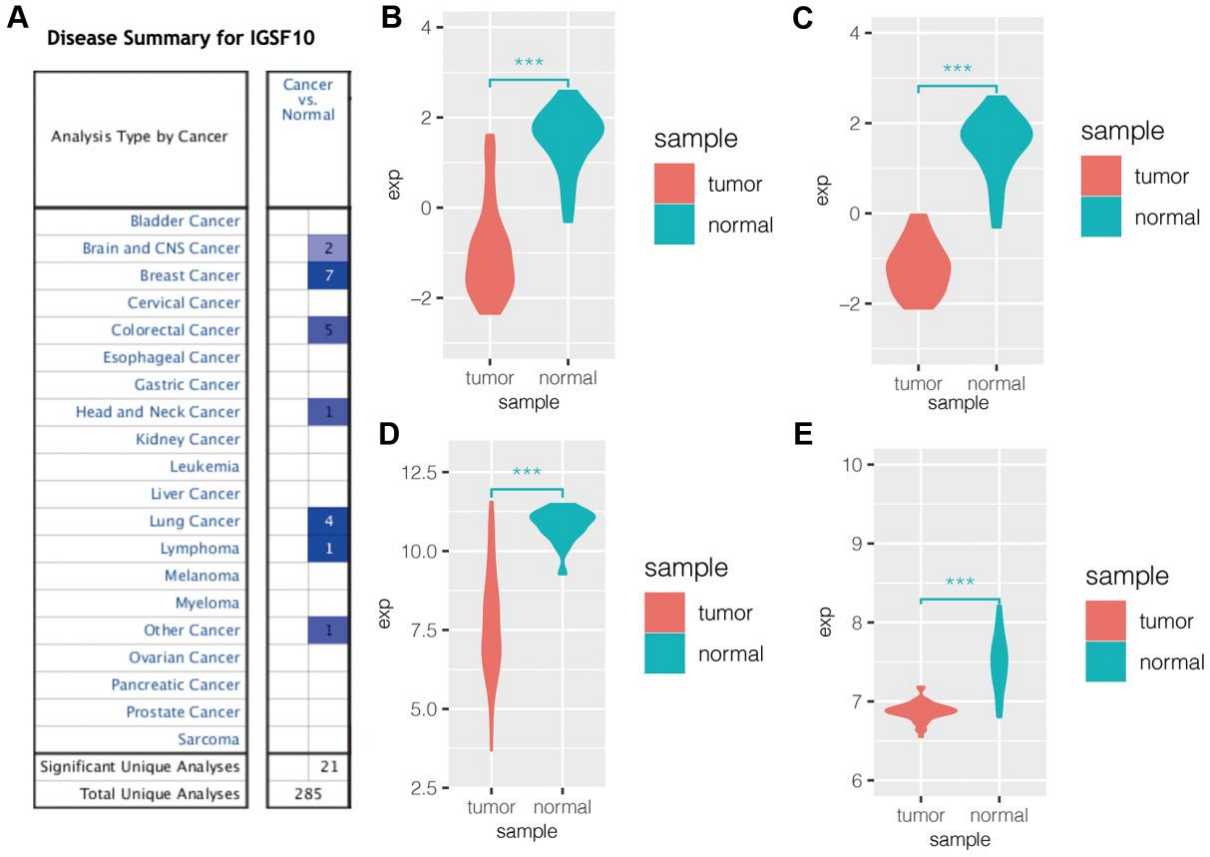
**IGSF10 expression is downregulated in several cancers including LUAD.** (**A**) Oncomine database analysis shows expression levels of IGSF10 in several cancer types. Blue represents low expression of IGSF10 in tumor tissues compared to the corresponding normal tissues; the numbers correspond to the datasets for each cancer type. (**B**) IGSF10 expression levels in LUAD and normal lung cancer samples in the GSE19188 dataset. (**C**) IGSF10 expression levels in LUSC and normal lung tissue samples in the GSE19188 dataset. (**D**) IGSF10 expression levels in LUAD and normal lung tissue samples in the GSE31210 dataset. (**E**) IGSF10 expression levels in LUAD and normal lung tissue samples in the GSE32863 dataset.

Oncomine database analysis showed that expression of IGSF10 was significantly reduced in lung cancer tissues compared to the corresponding normal lung tissues (Figure 1A). We then downloaded IGSF10 expression data in lung cancer datasets from the GEO database and observed downregulation of IGSF10 expression in the LUAD (Figure 1B) and LUSC (Figure 1C) samples from the GSE19188 dataset as well as lung cancer tissues in the GSE31210 and GSE32863 datasets (Figure 1D, 1E).

### Low IGSF10 expression correlates with poor survival outcomes in LUAD patients

**Figure 2:**
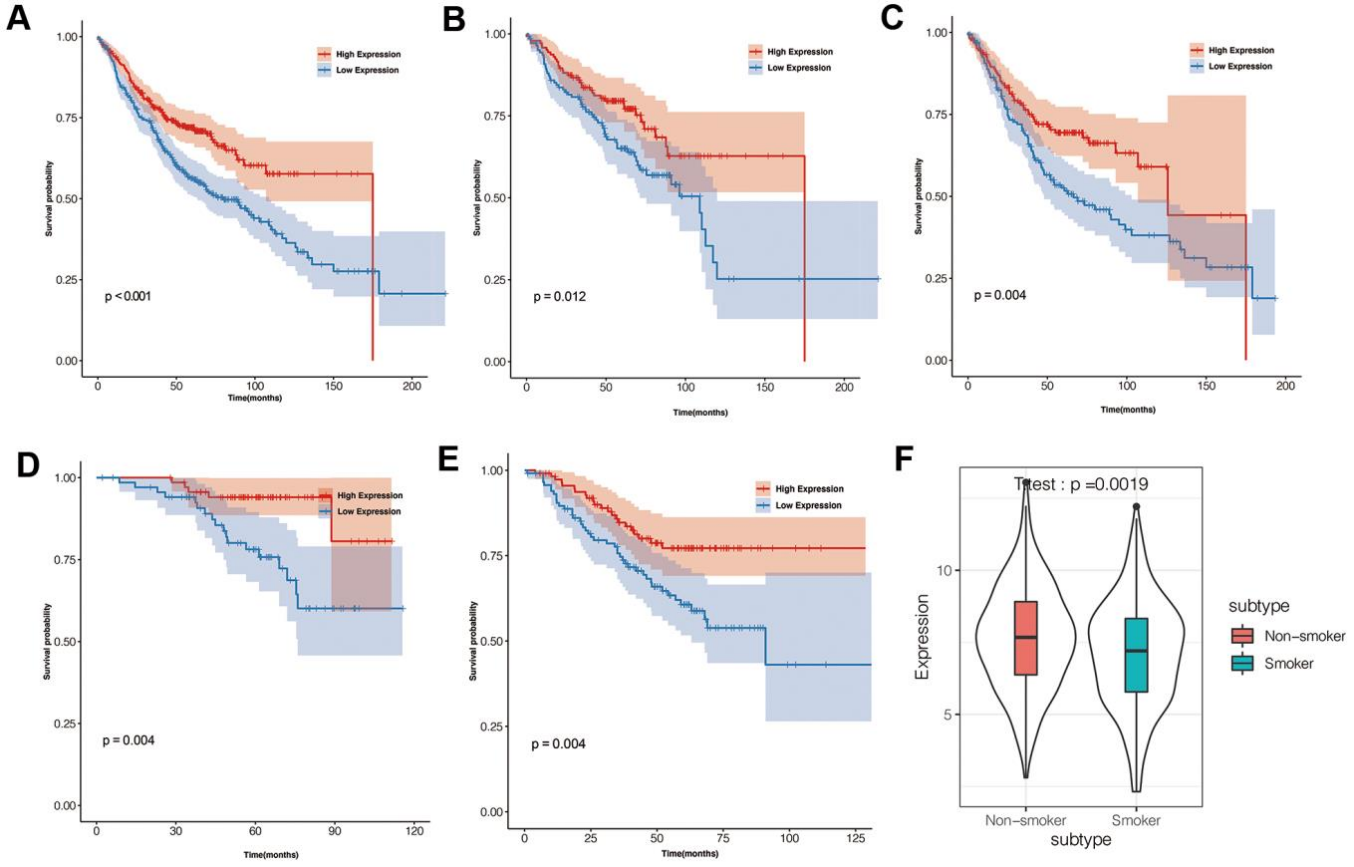
**Prognostic significance of IGSF10 expression levels in TCGA-LUAD dataset and clinical subgroups.** (**A**) Kaplan-Meier survival curve analysis shows overall survival (OS) rates of TCGA-LUAD dataset patients with high- and low- IGSF10 expression levels. (**B**) Kaplan-Meier survival curve analysis shows overall survival (OS) rates of female LUAD patients with high- and low-IGSF10 expression. (**C**) Kaplan-Meier survival curve analysis shows overall survival (OS) rates of male LUAD patients with high- and low-IGSF10 expression. (**D**) Kaplan-Meier survival curve analysis shows overall survival (OS) rates in non-smoker group LUAD patients with high- and low-IGSF10 expression. (**E**) Kaplan-Meier survival curve analysis shows overall survival (OS) rates of smoker group LUAD patients with high- and low-IGSF10 expression. (**F**) Spearman’s correlation analysis shows association between IGSF10 expression and smoking in LUAD patients from non-smoker and smoker subgroups.

LUAD patients were classified into high- and low-expression groups based on median IGSF10 expression. Kaplan-Meier survival analysis showed that overall survival of patients with low IGSF10 expression was significantly shorter than those with high IGSF10 expression (Figure 2A, *p*< 0.05). Moreover, survival outcomes of LUAD patients in clinicopathological categories such as females (Figure 2B, *p*< 0.05), males (Figure 2C, *p*< 0.05), non-smokers (Figure 2D, *p*< 0.05), and smokers (Figure 2E, *p* < 0.05) were associated with expression levels of IGSF10. Moreover, IGSF10 expression levels in LUAD patients were significantly higher in non-smokers compared to smokers (Figure 2F). We then performed univariate and multivariate Cox regression analysis to evaluate prognostic significance of IGSF10 expression, gender, stage, smoking status and age in the TCGA-LUAD data set. The results showed that IGSF10 and clinical stage were independent risk factors in LUAD patients (Table 1).

**Table 1:** Summary of univariate and multivariate analysis of clinicopathological parameters.

	**Univariate analysis**				**Multivariate analysis**			
	**HR**	**lower 95%CI**	**upper 95% CI**	**p-value**	**HR**	**lower 95%CI**	**upper 95%CI**	**p-value**
**IGSF10** high vs. low	0.7	0.52	0.95	0.021	0.66	0.49	0.9	0.01
**Gender** males vs. females	1.2	0.86	1.6	0.33	1.16	0.86	1.58	0.33
**Clinical stage** III/IV vs. I/II	2.5	1.8	3.4	3.1E-08	2.5	1.81	3.44	2.48E-08
**Smoking** Cat. 2,3,4,5 vs. Cat.1	0.91	0.6	1.4	0.64	0.86	0.56	1.32	0.49
**Age** >65 vs <=65	1.3	0.95	1.7	0.11	1.29	0.95	1.75	0.1

### Relationship between IGSF10 and proportion of tumor- infiltration immune cells in LUAD tissues

**Figure 3:**
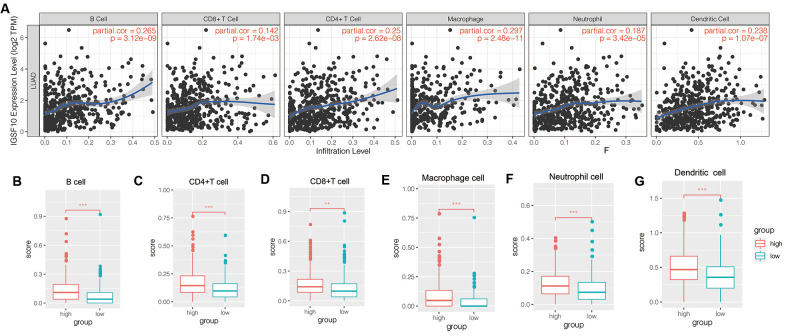
**Relationship between IGSF10 expression and tumor-infiltration of immune cells in TCGA-LUAD patient dataset.** (**A**) TIMER database analysis shows correlation between expression levels of IGSF10 and immunoscores corresponding to tumor-infiltrating immune cells in TCGA-LUAD patients. (**B**–**F**) TIMER database analysis shows relationship between IGSF10 expression levels and proportions of tumor-infiltrating immune cell types including (**B**) B cells; (**C**) CD4^+^ T cells; (**D**) CD8^+^ T cells; (**E**) Neutrophils; (**F**) Macrophages; and (**G**) Dendritic cells.

TIMER database analysis showed that IGSF10 expression positively correlated with the proportion of tumor-infiltrating B cells, CD4^+^ T cells, CD8^+^ T cells, neutrophils, macrophages, and dendritic cells in the TCGA-LUAD dataset (Figure 3A). Furthermore, tumor-infiltration levels of immune cells (B cells, CD4^+^ T cells, CD8^+^ T cells, neutrophils, macrophages, and dendritic cells) were significantly higher in patients with high IGSF10 expression compared to those with low IGSF10 expression (Figure 3B–3G).

### IGSF10 expression is significantly reduced in pan-cancer tissues and is inversely related to overall survival of patients belonging to six different cancer types

**Figure 4:**
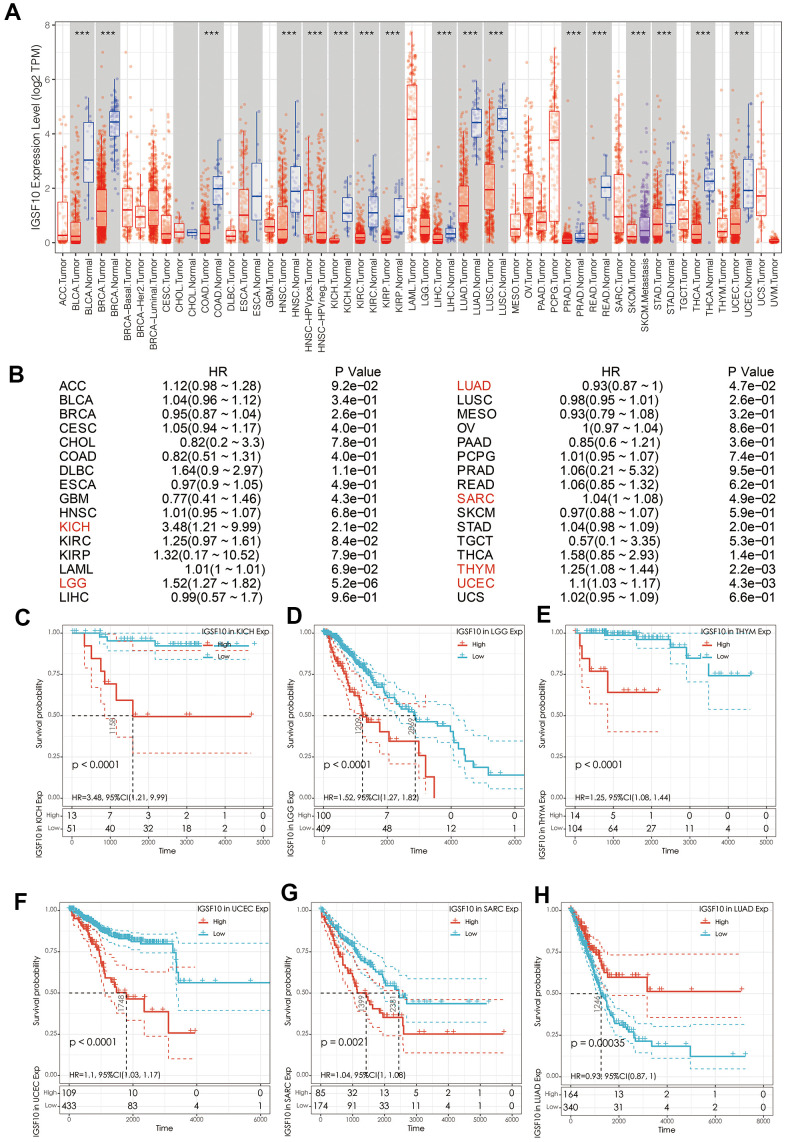
**Prognostic significance of IGSF10 expression in pan-cancer tissues.** (**A**) The expression levels of IGSF10 in pan-cancer and corresponding normal tissues from the TCGA database. (**B**–**H**) Survival curves show OS rates of high- and low-IGSF10 expressing tumor tissues from various TCGA pan-cancer datasets.

We then analyzed expression levels of IGSF10 in TCGA pan-cancer datasets. The expression of IGSF10 was significantly reduced in most cancer types (Figure 4A). Kaplan-Meier survival analysis showed that IGSF10 expression was inversely correlated with survival outcomes of kidney chromophobe (KICH), sarcoma (SARC), lower grade glioma (LGG), uterine corpus endometrial carcinoma (UCEC), thymoma (THYM), and LUAD patients (Figure 4B–4H).

### IGSF10 expression correlates with tumor infiltration of immune cells in multiple cancer types

**Figure 5:**
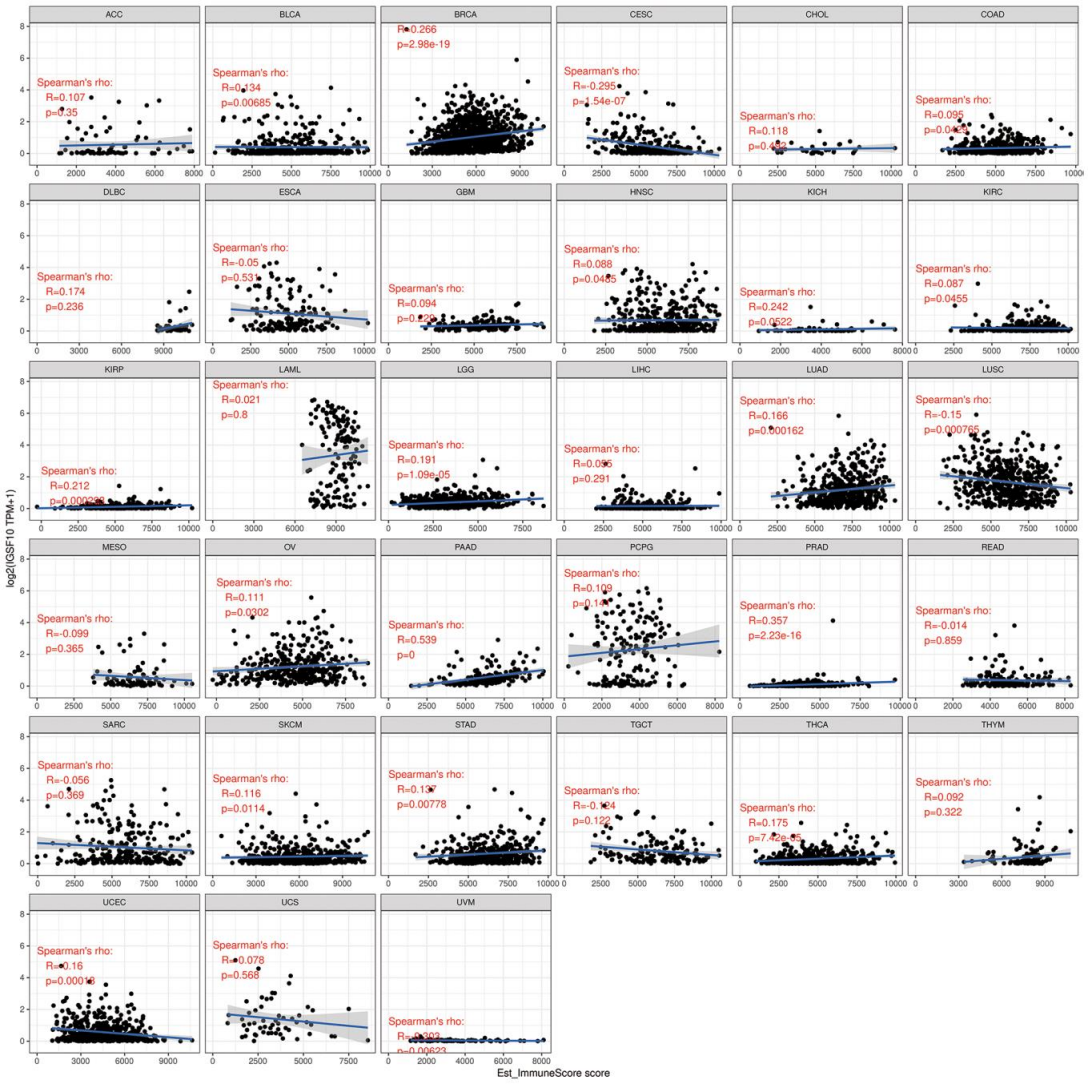
Correlation analysis between IGSF10 expression and immunoscores of pan-cancer tissues.

Previous studies have shown that tumor immune microenvironment plays an important role in tumor development and progression. Therefore, we analyzed the correlation between IGSF10 expression and immunoscores in 33 tumors. IGSF10 expression showed positive correlation with immunoscore in LUAD (R=0.166, P<0.001) and LUSC (R=0.15, P<0.001) (Figure 5). The highest correlation between IGSF10 expression and immunoscore was observed in pancreatic adenocarcinoma (PAAD) (R=0.539, P<0.05) (Figure 5). Overall, we observed significant correlation between IGSF10 expression and immunoscores in several cancer types (R=0.266, P=2.98e-19) (Figure 5).

### IGSF10 expression correlates with expression of several immune checkpoint genes (ICGs) and mismatch repair genes in several cancers

**Figure 6:**
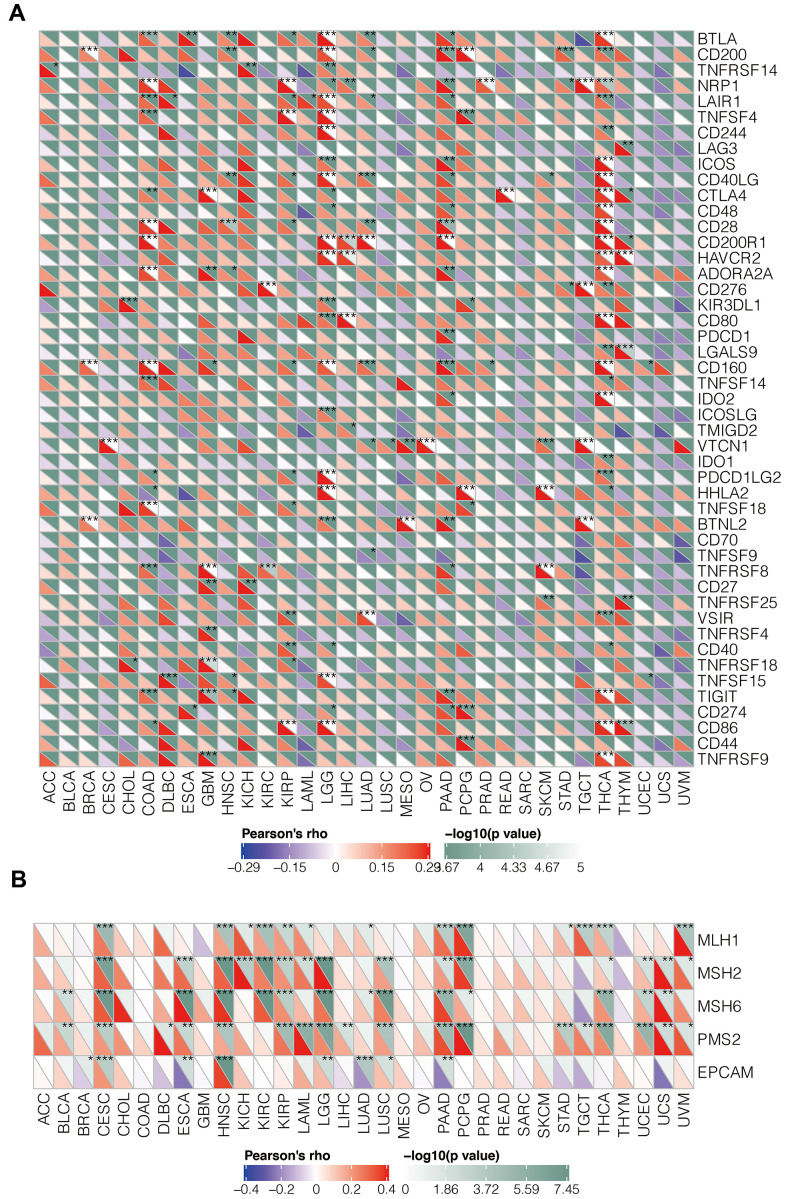
**Relationship between IGSF10 expression and levels of immune checkpoint genes (ICGs) as well as mis-match repair genes (MMRs) in pan-cancer tissues.** (**A**) The correlation analysis between IGSF 10 expression levels and immune checkpoint gene (ICG) expression in pan-cancer tissues. (**B**) Correlation analysis between IGSF 10 and five mismatch repair (MMR) genes, MLH1, MSH2, MSH6, PMS2, and EPCAM, in pan-cancer tissues. Note: * denotes p < 0.05; ** denotes p< 0.01; *** denotes p< 0.001.

We then analyzed association between IGSF10 expression and immune checkpoint genes in various tumors. Overall, we observed significant association between IGSF10 and immune checkpoint genes such as CD200R1, VSIR, CD160, CD28, BTLA, and CD40LG in several tumors including low-grade glioma (LGG) and thyroid carcinoma (THCA) (Figure 6A).

The loss of function of key mismatch repair (MMR) genes leads to accumulation of somatic mutations because of DNA replication errors. We performed correlation analysis between IGSF10 and five MMR genes (MLH1, MSH2, MSH6, PMS2, and EPCAM) and observed negative correlation between IGSF10 and EPCAM in LUAD, and significant positive correlation between IGFS10 and MMRs in Cervical squamous cell carcinoma (CESE), pancreatic adenocarcinoma (PAAD), and head and neck squamous cell carcinoma (HNSC) (Figure 6B).

### Functional enrichment analysis of differentially expressed genes in TCGA-LUAD dataset

**Figure 7:**
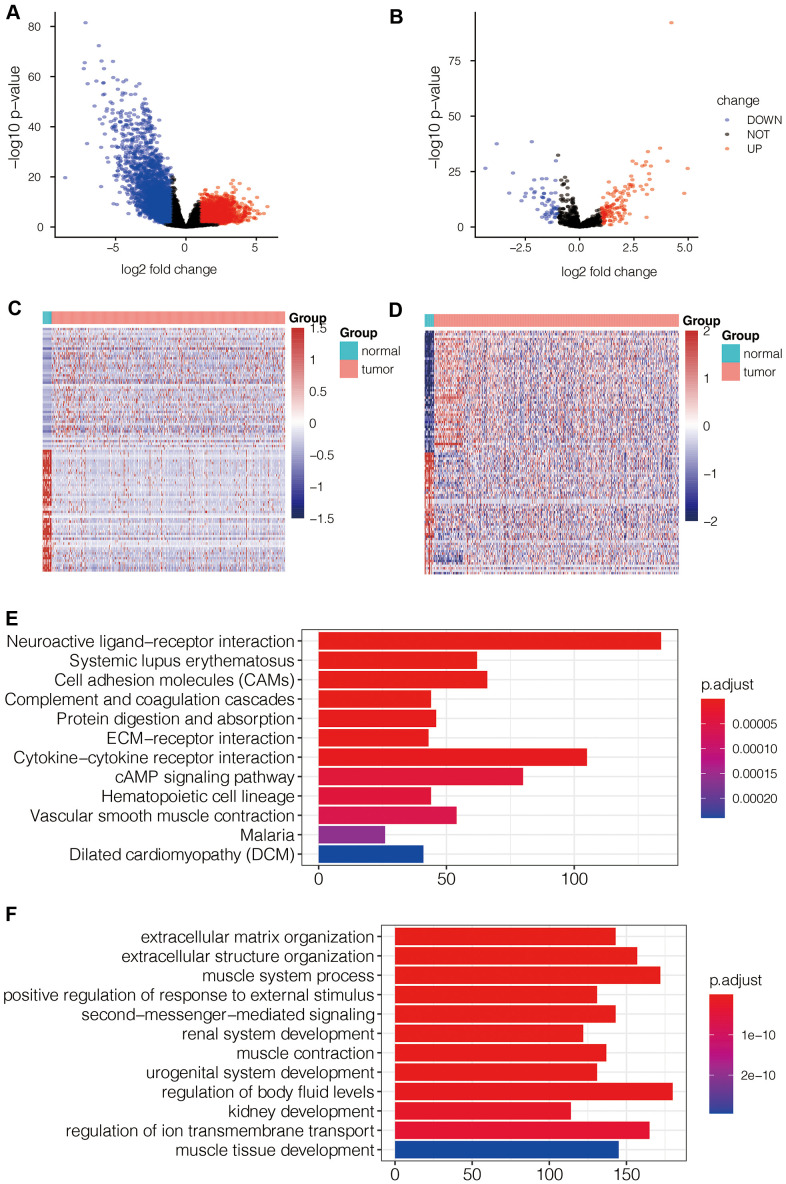
**Identification of differentially expressed mRNAs and miRNAs in LUAD tissues and their functional enrichment analysis.** (**A**) Volcano map shows differentially expressed mRNAs (DEG- mRNAs) in the TCGA-LUAD dataset. (**B**) Volcano map shows differentially expressed miRNAs (DEG- miRNAs) in the TCGA-LUAD dataset. (**C**) Heat map shows expression levels of DEG- mRNAs in TCGA-LUAD dataset. (**D**) Heat map shows expression levels of DEG- miRNAs in TCGA-LUAD dataset. (**E**) KEGG pathway analysis of DEG- mRNAs shows the top 12 upregulated pathways in LUAD tissues of the TCGA dataset. (**F**) Gene ontology analysis of DEG-mRNAs shows the top 12 biological processes in LUAD tissues of the TCGA dataset.

RNA sequencing analysis of the TCGA-LUAD dataset identified 5771 differentially expressed mRNAs (DEG-mRNAs) including 2367 up-regulated and 3404 down-regulated mRNAs (Figure 7A) and 199 differentially expressed miRNAs (DEG-miRNAs) including 139 up-regulated and 60 down-regulated DEG-miRNAs (Figure 7B). The heat maps of DEG-mRNAs and DEG-miRNAs are shown in Figure 7C, 7D.

Functional enrichment analysis of the DEG-mRNAs that pathways such as cell adhesion molecules (CAMs), ECM-receptor interactions, cAMP signaling pathway, and other tumor related pathways were significantly enriched in LUAD tissues (Figure 7E). The top biological processes represented by the DEG-mRNAs included positive regulation of response to external stimulus and extracellular matrix organization (Figure 7F).

### Identification of miRNAs that regulate IGSF10 in LUAD tissues

We further investigated potential mechanisms underlying the down-regulation of IGSF10 in LUAD tissues. MicroRNAs are small non-coding RNAs that downregulate expression level of their target genes by binding to complementary sequence in the 3'-UTRs of the target mRNAs. Therefore, we used the miRwalk2.0 database to identify potential miRNAs that interact with the 3’UTR of IGSF10. We identified hsa-miR-17-5p, hsa-miR-106a-5p, hsa-miR-106b-5p, hsa-miR-93-5p, and hsa-miR-20a-5p as potential candidates (Supplementary Figure 1A). Then, we classified LUAD patients into high- and low-expression groups based on the median expression levels of the candidate IGSF10-targeting miRNAs. Kaplan-Meier survival curves showed that overall survival rates of LUAD patients with low expression of hsa-miR-106b-5p was significantly higher compared to those of patients with high hsa-miR-106b-5p expression (Supplementary Figure 1B, p = 0.032). The other miRNAs did not show prognostic significance (Supplementary Figure 1C–1F). Therefore, we selected hsa-miR-106b-5p for further analysis.

### Knockdown of miR-106b-5p inhibits proliferation and progression of LUAD cells

**Figure 8:**
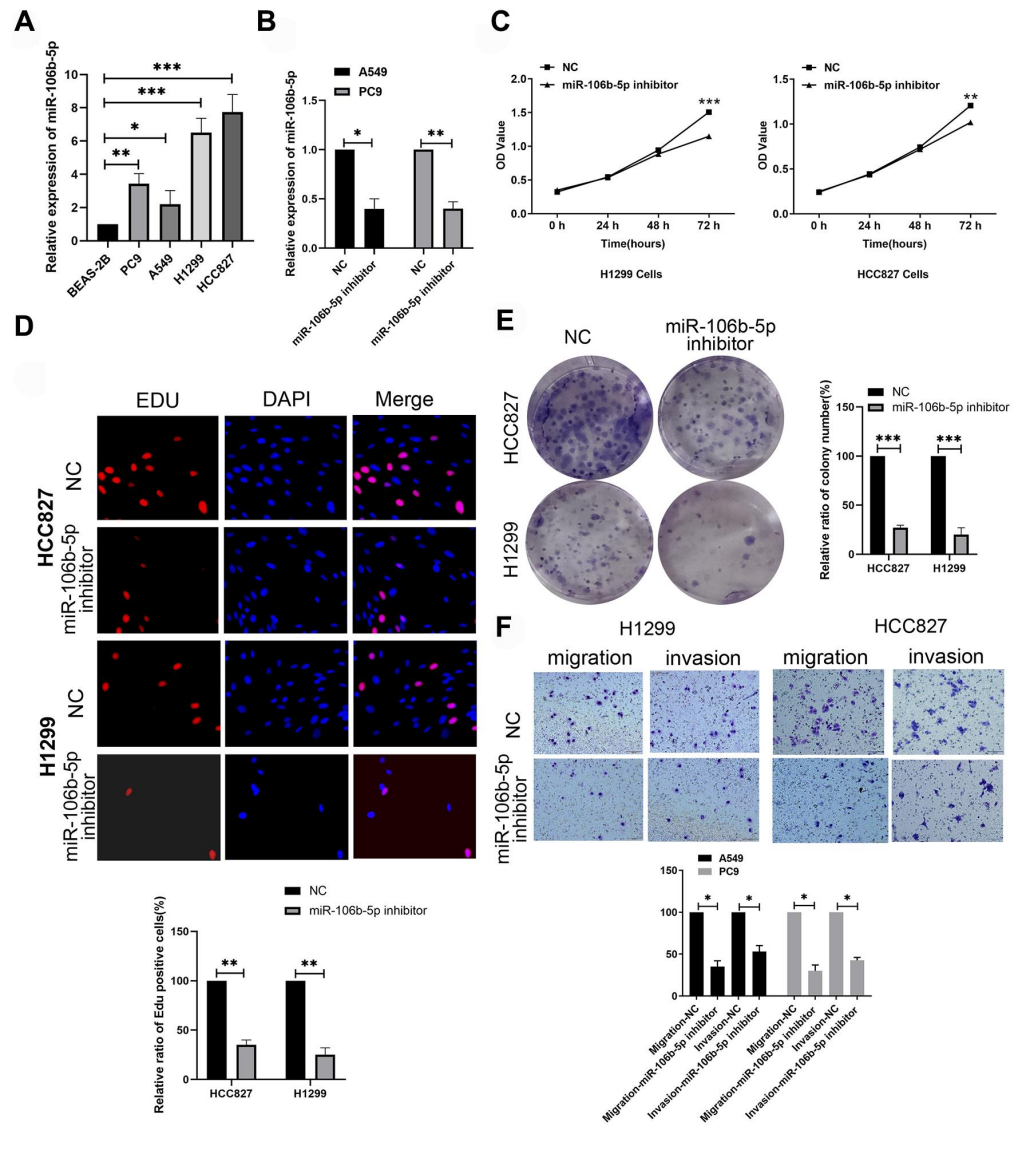
**MiR-106b-5p promotes proliferation and progression of lung cancer cells.** (**A**) RT-qPCR analysis shows expression levels of miR-106b-5p in the human bronchial epithelial cell line, BEAS-2B, and LUAD cell lines, H1299, HCC827, A549 and PC9. (**B**) RT-qPCR analysis shows miR-106b-5p expression levels in H1299 and HCC827 cells transfected with negative control (NC) or miR-106b-5p inhibitor. (**C**) MTT assay shows proliferation status of H1299 and HCC827 cells transfected with negative control (NC) or miR-106b-5p inhibitor. (**D**) EdU assay results show proliferation status of H1299 and HCC827 cells transfected with negative control (NC) or miR-106b-5p inhibitor. (**E**) Colony formation assay shows the number of colonies formed by H1299 and HCC827 cells transfected with negative control (NC) or miR-106b-5p inhibitor. (**F**) Transwell assays show migration and invasion ability of H1299 and HCC827 cells transfected with negative control (NC) or miR-106b-5p inhibitor. Results are represented as means ± SD; ^*^p < 0.05, ^**^p < 0.01, and ^***^p < 0.001 vs. control group.

Next, we analyzed the expression levels of miR-106b-5p in the human bronchial epithelial cell line, BEAS-2B, and LUAD cell lines, H1299, HCC827, A549 and PC9 using RT-qPCR. The expression levels of miR-106b-5p were significantly higher in all LUAD cell lines compared to the human bronchial epithelial cell line, BEAS-2B (Figure 8A). The levels of miR-106b-5p were highest in H1299 and HCC827 cell lines. RT-qPCR analysis showed that miR-106b-5p levels were significantly reduced in miR-106b-5p inhibitor-transfected H1299 and HCC827 cells compared to the corresponding controls (Figure 8B). MTT, EdU, and colony formation assays showed that proliferation of miR-106b-5p inhibitor-transfected H1299 and HCC827 cells were significantly reduced compared to the corresponding controls (Figure 8C–8E). Transwell invasion and migration assays demonstrated that miR-106b-5p knockdown significantly reduced invasiveness and migration of H1299 and HCC827 cells (Figure 8F). These results demonstrated that miR-106b-5p promoted proliferation and progression of LUAD cells.

### MiR-106b-5p suppresses IGSF10 expression in LUAD cells by directly binding to its 3’-UTR

**Figure 9:**
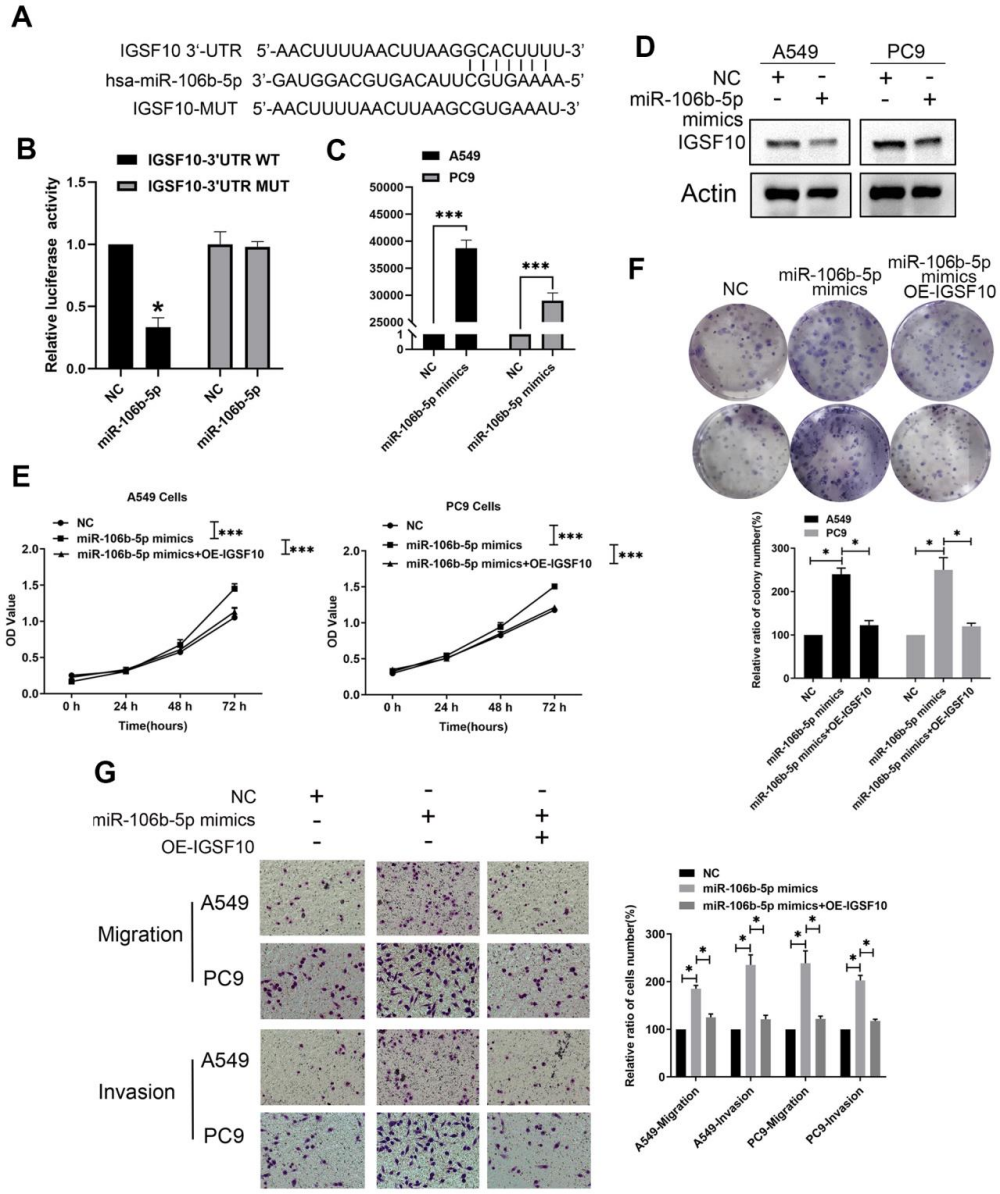
**MiR-106b-5p suppresses IGSF10 expression by binding to its 3’-UTR.** (**A**) Dual luciferase assay results show that miR-106b-5p directly targets the 3′-UTR of IGSF10. (**B**) Dual luciferase assay results show that relative luciferase activity is significantly repressed in LAUD cells co-transfected with miR-106b-5p and wild type 3′-UTR of IGSF10 compared to those co-transfected with miR-106b-5p and mutated 3′-UTR of IGSF10 (P < 0.05). (**C**) RT-qPCR analysis shows expression levels of miR-106b-5p in A549 and PC9 cells transfected with non-specific control (miR-NC) or miR-106b-5p mimics. (**D**) Western blot analysis shows IGSF10 protein levels in A549 and PC9 cells transfected with non-specific control (miR-NC) or miR-106b-5p mimics. (**E**) MTT assay and (**F**) colony formation assays show proliferative ability of A549 and PC9 cells transfected with non-specific control (miR-NC) or miR-106b-5p mimics. (**G**) Transwell assay results show migration and invasion ability of A549 and PC9 cells transfected with non-specific control (miR-NC) or miR-106b-5p mimics. Results are shown as means ± SD; ^*^ p < 0.05, ^**^p < 0.01, and ^***^p < 0.001 vs. control group.

We then studied the regulatory relationship between miR-106b-5p and IGSF10. Dual luciferase reporter assays demonstrated that relative luciferase activity was significantly decreased in LUAD cells co-transfected with miR-106b-5p and luciferase vector containing wild type 3′-UTR of IGSF10 compared to the corresponding controls (P < 0.05) (Figure 9A, 9B). We then overexpressed miR-106b-5p in A549 and PC9 cells by transfecting them with miR-106b-5p mimics (Figure 9C). MiR-106b-5p-overexpressing A549 and PC9 cells showed reduced expression of IGSF10 compared to the corresponding controls (Figure 9D). These results confirmed that miR-106b-5p suppressed IGSF10 expression in LUAD cells by binding to its 3'-UTR.

Next, we investigated if miR-106b-5p regulated the malignant phenotype of LUAD cells via IGSF10. MiR-106b-5p-overexpressing LUAD cells showed significantly higher rates of proliferation (Figure 9E, 9F) as well as migration and invasion (Figure 9G) compared to the controls. However, these effects were reversed by overexpressing IGSF10 in miR-106b-5p mimic-transfected LUAD cells (Figure 9E, 9G). These results confirmed that miR-106b-5p regulated growth and progression of LUAD cells via IGSF10.

## DISCUSSION

Lung cancer includes diverse subtypes with significant differences in molecular, pathological, clinical and prognostic characteristics [21]. Currently, there is an urgent need to characterize new biomarkers that can accurately predict prognosis and improve personalized therapy for lung cancer patients [22]. IGSF10 regulates early migration of GNRH (gonadotropin-releasing hormone) neurons and mutations in IGSF10 are implicated in delayed puberty [10, 23, 24]. Moreover, IGSF10 mutations are associated with endometrial cancer [25] and gastric cancer [26]. Furthermore, downregulation of IGSF10 is associated with radiation-induced bone tumorigenesis [27]. Our previous study demonstrated that IGSF10 functions as a tumor suppressor in lung cancer via integrin-β1/FAK signaling pathway [9]. In this study, we showed that IGSF10 expression was significantly reduced in multiple cancer types. Survival analysis showed that IGSF10 expression correlated with overall survival of patients belonging to 6 cancer types, namely, KICH, SARC, LGG, UCEC, THYM, and LUAD. Moreover, our analysis showed that IGSF10 was an independent prognosis factor for LUAD patients.

TIMER database analysis showed that IGSF10 expression positively correlated with the proportion of B cells, CD4^+^ T cells, CD8^+^ T cells, macrophages, neutrophils, and dendritic cells in LUAD tissues. Among these, B cells, CD4^+^ T cells and CD8^+^ T cells play a central role in adaptive immunity through expression of neoantigens and HLA class I molecules on their cell surface, which enables recognition and killing of lung cancer cells [28, 29]. Neutrophils, macrophages and dendritic cells are integral members of innate immunity [30]. This suggests that IGSF10 regulates both adaptive and innate immune responses in lung cancer. We also observed positive correlation between IGSF10 and immunoscore in LUAD tissues. Tumor-infiltrating immune cells play a key role in immune surveillance and destruction of tumor cells [31]. Our results showed that LUAD patients with high IGSF10 expression correlated with increased levels of tumor-infiltrating immune cells. These results suggest that IGSF10 modulates the tumor microenvironment. Taken together, our results showed that IGSF10 is a potential prognostic biomarker for LUAD.

MiRNAs are small endogenous non-coding RNAs that inhibit target mRNA translation by binding to their 3'-UTRs. In this study, we identified negative correlation between miR-106b-5p and IGSF10. MiR-106b-5p is upregulated in gastric cancer [32], hepatocellular carcinoma [33], and glioma [34] and acts as an oncogenic miRNA. MiR-106b-5p also acts as a tumor suppressor in breast cancer [35] and ovarian cancer [36]. However, the role of miR-106b-5p is controversial in lung cancer. Previous reports suggest that it functions both as an oncogene and as a tumor suppressor [19, 20]. We analyzed expression of miR-106b-5p in normal bronchial epithelial cells and LUAD cell lines and found that miR-106b-5p was over-expressed in LUAD cells. Furthermore, inhibition of miR-106b-5p significantly decreased proliferation, migration, and invasiveness of LUAD cells. These results demonstrated that miR-106b-5p promoted growth and progression of LUAD cells.

Previous studies demonstrated that miR-106b-5p regulated the expression of PTTG1 [37], LIMK1 [38], TIMP2 [39], and NOR1 [40]. However, the relationship between miR-106b-5p and IGSF10 was not reported previously. In this study, dual luciferase reporter assays confirmed that miR-106b-5p directly binds to the 3’UTR of miR-106b-5p in LUAD cells. Furthermore, miR-106b-5p promoted proliferation, migration, and invasion of LUAD cells by inhibiting expression of IGSF10.

However, there are some limitations in our study. For example, our study was based on *in vitro* experiments and bioinformatics analysis, and lacked *in vivo* experiments. Hence, future studies are necessary to confirm our findings.

In summary, our study demonstrated that IGSF10 expression was an independent prognostic factor in LUAD. Moreover, we demonstrated that miR-106b-5p promoted proliferation, migration, and invasion of LUAD cells by suppressing the expression of IGSF10. Therefore, IGSF10 and miR-106b-5p are potential prognostic biomarkers and therapeutic targets in LUAD.

## MATERIALS AND METHODS

### Data download and preprocessing

We downloaded miRNA expression data for 508 LUAD and 45 para-cancerous lung tissue samples (TCGA-LUAD cohort) from the UCSC Xena database. We also downloaded mRNA expression data for 513 LUAD and 59 para-cancerous lung tissue samples from TCGA biolinks. We then combined the two datasets and obtained complete clinical information for the 521 study samples including 503 LUAD and 19 para-cancerous lung tissue samples. The clinical information of the LUAD patients included in this study is shown in Table 2.

**Table 2:** Clinical information of TCGA-LUAD patients.

**Covariates**	**Category**	**Number**
**Sex**	Females	269
	Males	234
**Stages**	I	271
	II	118
	III	83
	IV	24
	NA	7
**Smokers**	1	72
	2	120
	3	133
	4	160
	5	4
**Age**	<=65	236
	>65	248

### Identification of differentially expressed RNAs and functional enrichment analysis

Limma R package [41] was used to analyze mRNA and miRNA expression data of LUAD and para-cancerous lung tissue samples to identify differentially expressed mRNAs and miRNAs with logFC | > 1 and p value <0.05 as cutoff parameters. Moreover, volcano plots and heat maps were constructed using ggplot2 package [42] and pheatmap package [43], respectively.

### Construction of miRNA-mRNA interaction networks

We used miRWalk (http://zmf.umm.uni-heidelberg.de/apps/zmf/mirwalk2/) database to analyze differentially expressed miRNAs and mRNAs. The miRWalk database integrates information from miRDB, TargetScan, miRTarBase, and other databases for miRNA target genes from human, mouse and other species. We used Cytoscape software (https://cytoscape.org/) to construct interaction network between negatively correlating miRNAs and mRNAs that are differentially expressed in LUAD tissues.

### Expression and prognostic analysis of IGSF10 expression in pan-cancer tissues

We downloaded transcriptome data of 33 cancer types from the UCSC Xena database and all normal tissues from the GTEx portal. We analyzed differential expression of IGSF10 in the various cancer types and their corresponding normal tissues and constructed a box diagram. We then analyzed RNA-seq data from GEO and TCGA databases using KM-plotter and performed KM survival curve analysis for various cancer types including lung cancer, breast cancer, and others by classifying patients into high- and low-IGSF10 expression groups using median IGSF10 expression as the cut-off value. We also performed subgroup analysis for various clinicopathological parameters such as gender, age, and smoking.

### Correlation analysis between IGSF10 expression levels and proportions of tumor-infiltrating immune cells

We performed TIMER database analysis to determine estimate scores for tumor-infiltrating immune cells, namely, B cells, CD4^+^ T cells, CD8^+^ T cells, neutrophils, macrophages, and dendritic cells, for 33 different cancer types. Spearman correlation analysis was performed to determine the association between *IGSF10* gene expression and tumor-infiltration of immune cells based on the immunoscores. The samples were grouped according to IGSF10 expression levels and their estimate scores were evaluated using boxplots. The differences between groups were compared using Student's t-test.

### Cell culture and transfections

Human bronchial epithelial cells (BEAS-2B) and LUAD cell lines (H1299, HCC827, A549 and PC9) were cultured in RPMI-1640 medium supplemented with 10% FBS, at 37° C and 5% CO_2_. LUAD cells were transfected with miR-106b-5p mimics, miR-106b-5p inhibitors, their corresponding controls, and IGSF10-overexpression plasmids using JetPRIME® (Polyplus Transfection, New York, USA) according to the manufacturer's instructions at indicated doses and time points.

### LUAD cell malignant phenotype assay

MTT and Transwell assays were performed as previously described [9].

### Dual luciferase reporter assay

A549 cells were co-transfected with 0.5 μg firefly luciferase reporter plasmids, 0.005 μg pRL-TK luciferase control vector, and 50nM miR-106b-5p or miR-NC in 24-well plates. The relative luciferase assays were performed at 24 h after transfection using the dual luciferase reporter assay system (Promega, Madison, WI, USA) according to the manufacturer's protocol.

### Real-time quantitative PCR

Total RNA was purified from formalin-fixed, paraffin-embedded tissue sections using miRNeasy FFPE Kit (Qiagen, USA) according to manufacturer's protocol. Then, equal amounts of RNA samples were analyzed by qRT-PCR according to the manufacturer's instructions.

### Statistical analysis

All data are represented as means ± standard deviation (S.D) based on at least three independent experiments. Statistical analysis was performed using GraphPad Prism 7.0 software (GraphPad software, La Jolla, CA). P-values < 0.05 were considered statistically significant.

## Supplementary Material

Supplementary Figure 1
