# Supplementary material for: MicroRNA-106b-5p inhibits growth and progression of lung adenocarcinoma cells by downregulating IGSF10
Source: Aging (Albany NY). 2021 Jul 29;13(14):18740–56. doi: 10.18632/aging.203318 (PMC8351668; doi:10.18632/aging.203318)
Supplement: Supplementary Figure 1 [file aging-13-203318-s001.pdf]

SUPPLEMENTARY FIGURE

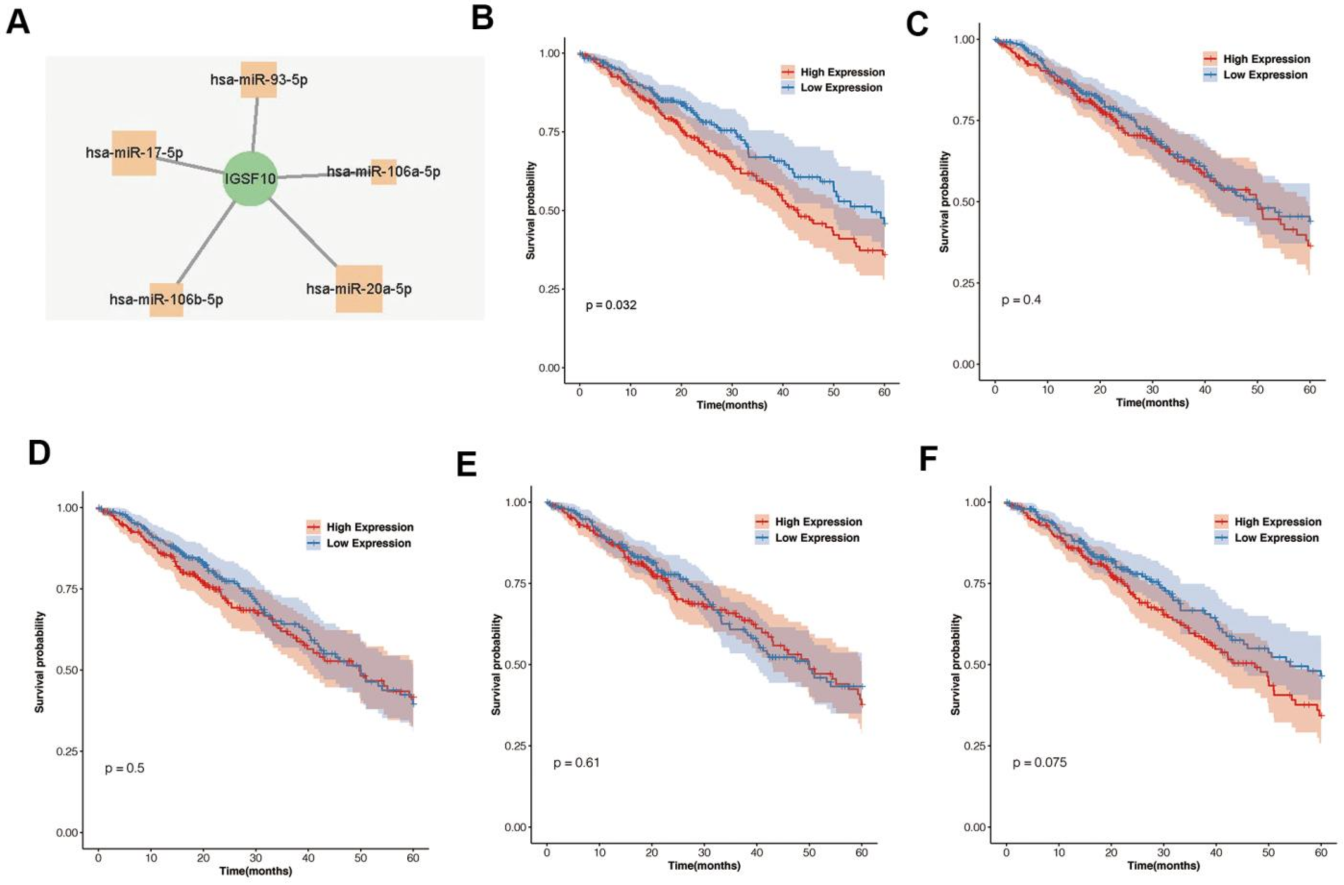

Supplementary Figure 1. Bioinformatics and survival analysis of potential IGSF10-binding miRNAs. (**A**) MiRwalk2.0 database analysis shows miRNAs that potentially bind to the 3'-UTR of IGSF10. (**B–F**) Kaplan-Meier survival curves show overall survival of LUAD patients with high- and low-expression levels of hsa-miR-17-5p, hsa-miR-106a-5p, hsa-miR-106b-5p, hsa-miR-93-5p, and hsa-miR-20a-5p, all of which potentially bind to the 3'UTR of IGSF10. The abscissa represents survival time; the ordinate represents survival probability.
